# Supplementary material for: MYCBP interacts with Sakura and Otu and is essential for germline stem cell renewal and differentiation and oogenesis
Source: bioRxiv. 2025 Jul 2:2025.07.01.662550. Preprint. [Version 1] doi: 10.1101/2025.07.01.662550 (PMC12236646; doi:10.1101/2025.07.01.662550)

bioRxiv preprint doi: https://doi.org/10.1101/2025.07.01.662550; this version posted July 2, 2025. The copyright holder for this preprint (which was not certified by peer review) is the author/funder, who has granted bioRxiv a license to display the preprint in perpetuity. It is made available under aCC-BY 4.0 International license.

Supplemental Figure Legends

Fig S1. MYCBP and Sakura form homodimers and MYCBP binds Myc-Otu, but not 3xMyc-CycA, Myc-Bam, 3xMyc-EGFP, or dMyc

Co-immunoprecipitation using S2 cell lysates and anti-FLAG (A, C, D) or anti-HA beads (D) followed by Western blotting.

Fig S2. Alphafold-predicted structures.

Protein complex structures predicted using Alpha-fold. full-length MYCBP, Sakura, and human MYCBP are used while Otu(N) is 1-405 aa region and OTUD4(N) is 1–350 aa region.

Fig S3. In vitro deubiquitination assay

Fluorescence intensity. Mean ± SD (n = 3). Firefly Luciferase was used as a negative control.

Fig S4. Male fertility assay

bioRxiv preprint doi: https://doi.org/10.1101/2025.07.01.662550; this version posted July 2, 2025. The copyright holder for this preprint (which was not certified by peer review) is the author/funder, who has granted bioRxiv a license to display the preprint in perpetuity. It is made available under aCC-BY 4.0 International license.

(A) Confocal images of the apical tip region of a testis from a *mycbp-EGFP* transgenic fly. MYCBP-EGFP (green), Vasa (red), and DAPI (blue). Hub cells are marked with yellow star. Scale bar: 20 μm.

(B) Numbers of progeny obtained from crosses between test males and wild-type (OregonR) virgin females. Mean ± SD (n = 5).

Fig S5. *mycbp^{null}* ovaries are tumorous

Violin plots showing the number of GSC-like cells per germaria in 2-5-day-old flies. Mean ± SD. P-value < 0.001 (Student's t-test, unpaired, two-tailed) is indicated by ***.

Fig S6. *mycbp^{null}* germline clones intrinsically cause tumorous phenotypes

Number of unmarked (GFP-positive) GSC-like cells in germaria containing marked (GFP-negative) GSCs at 4, 7, and 14 days after clone induction.

bioRxiv preprint doi: https://doi.org/10.1101/2025.07.01.662550; this version posted July 2, 2025. The copyright holder for this preprint (which was not certified by peer review) is the author/funder, who has granted bioRxiv a license to display the preprint in perpetuity. It is made available under aCC-BY 4.0 International license.

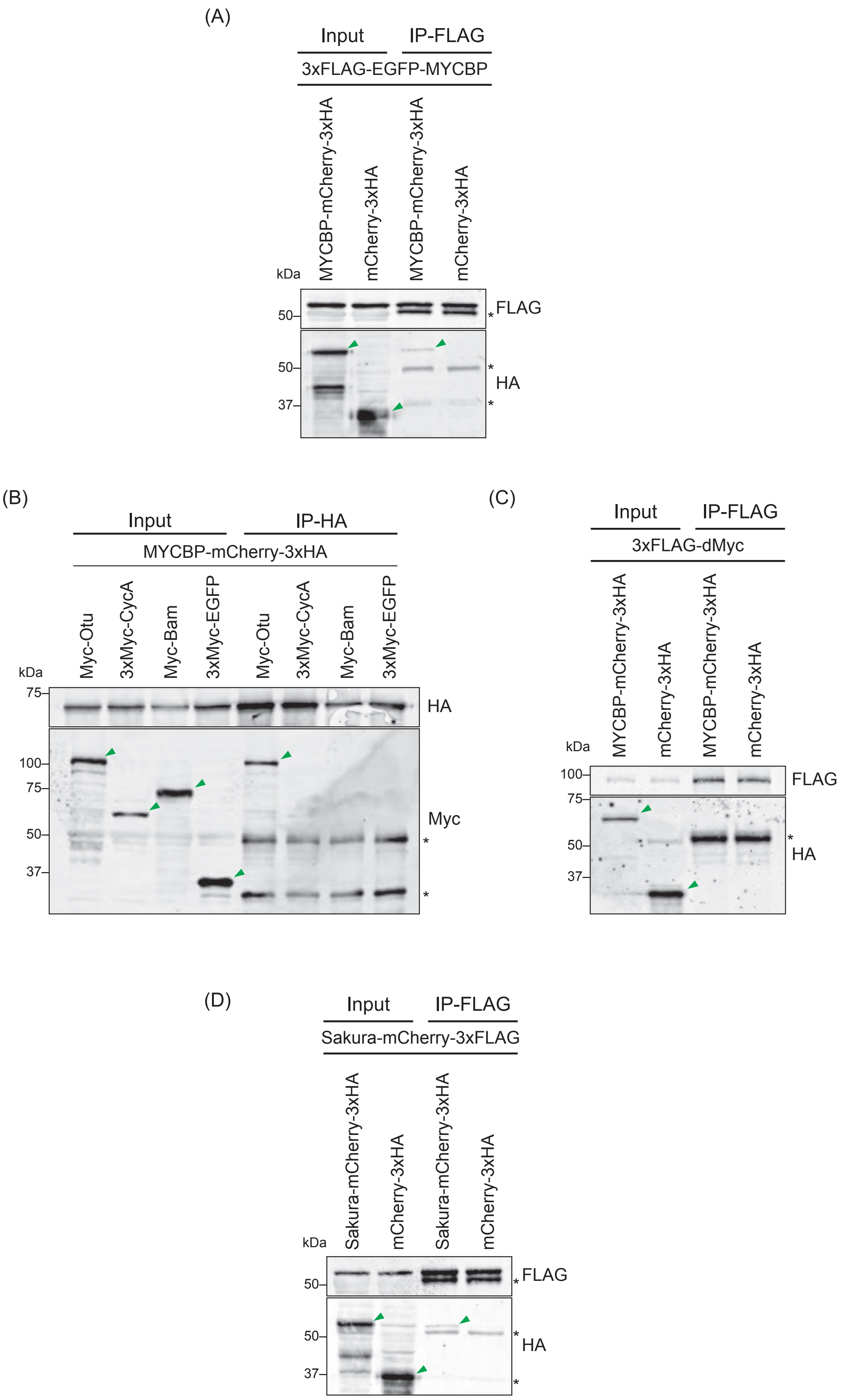

bioRxiv preprint doi: https://doi.org/10.1101/2025.07.01.662550; this version posted July 2, 2025. The copyright holder for this preprint (which was not certified by peer review) is the author/funder, who has granted bioRxiv a license to display the preprint in perpetuity. It is made available under aCC-BY 4.0 International license.

Figure S2. Azlan et al.

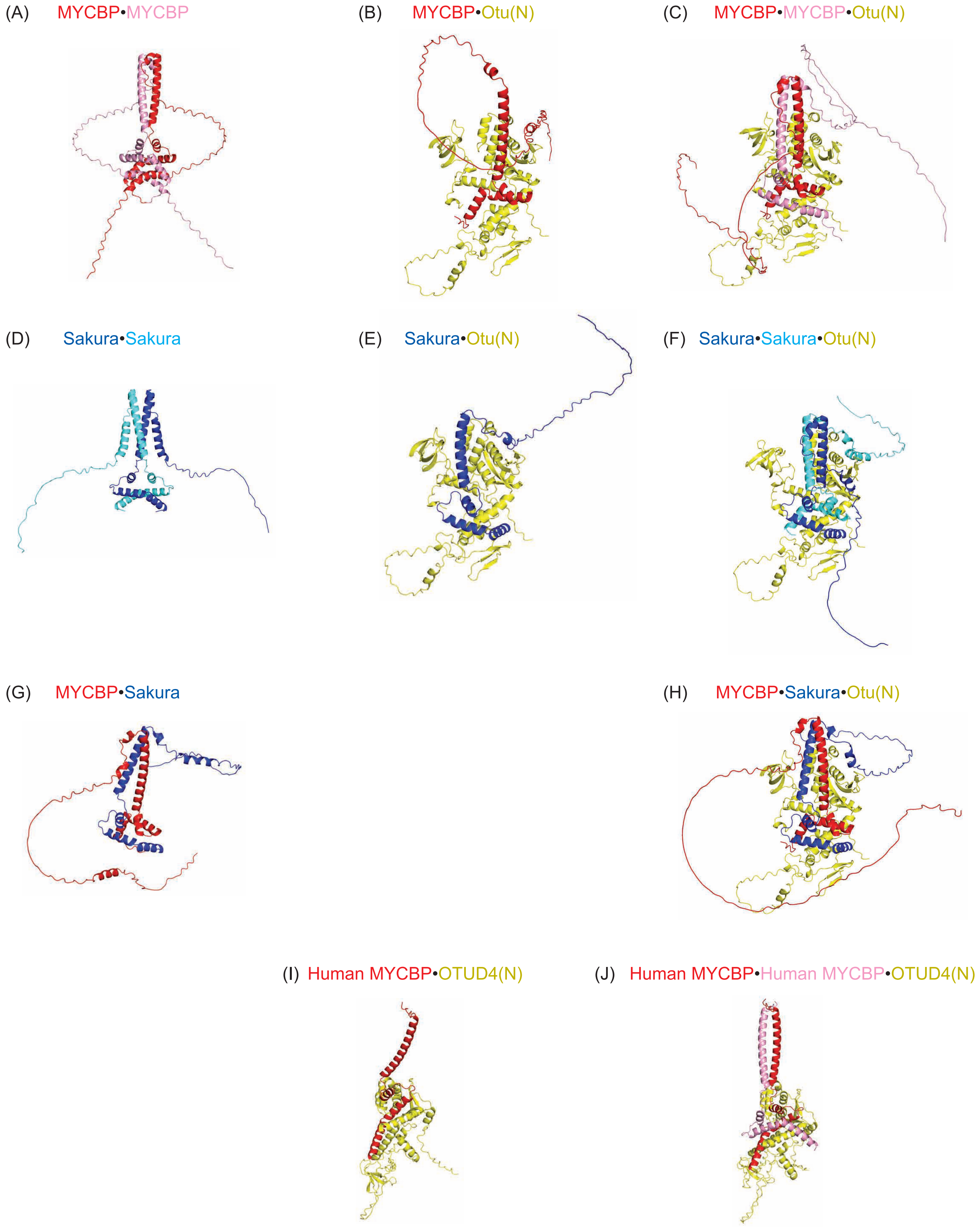

bioRxiv preprint doi: https://doi.org/10.1101/2025.07.01.662550; this version posted July 2, 2025. The copyright holder for this preprint (which was not certified by peer review) is the author/funder, who has granted bioRxiv a license to display the preprint in perpetuity. It is made available under aCC-BY 4.0 International license.

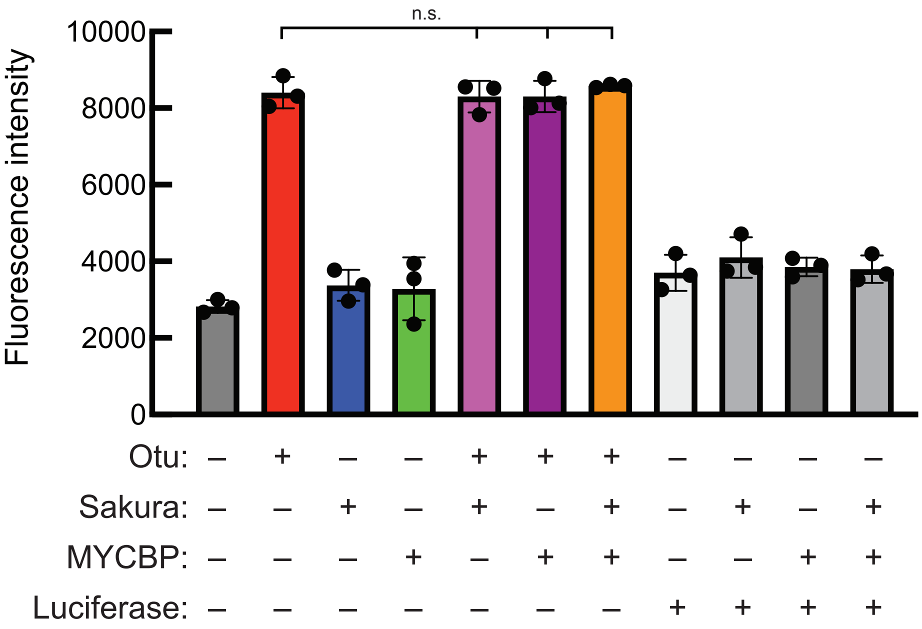

bioRxiv preprint doi: https://doi.org/10.1101/2025.07.01.662550; this version posted July 2, 2025. The copyright holder for this preprint (which was not certified by peer review) is the author/funder, who has granted bioRxiv a license to display the preprint in perpetuity. It is made available under aCC-BY 4.0 International license.

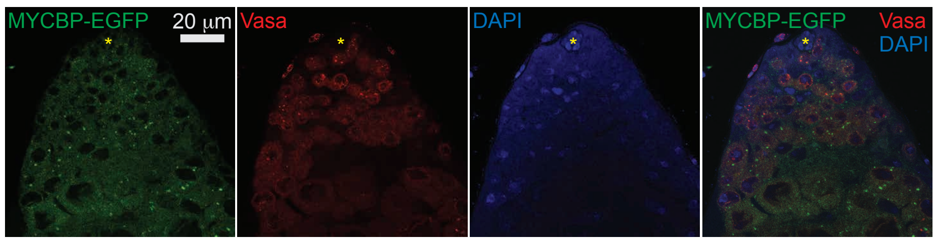

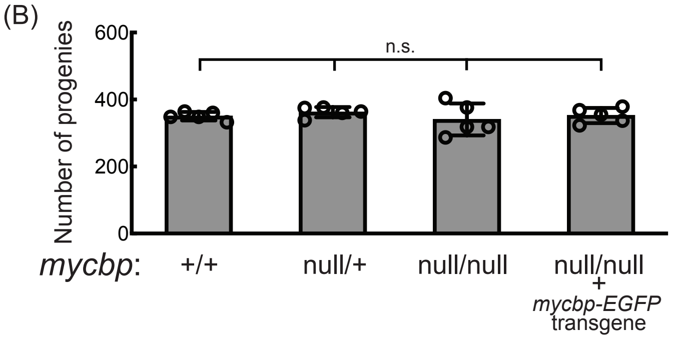

bioRxiv preprint doi: https://doi.org/10.1101/2025.07.01.662550; this version posted July 2, 2025. The copyright holder for this preprint (which was not certified by peer review) is the author/funder, who has granted bioRxiv a license to display the preprint in perpetuity. It is made available under aCC-BY 4.0 International license.

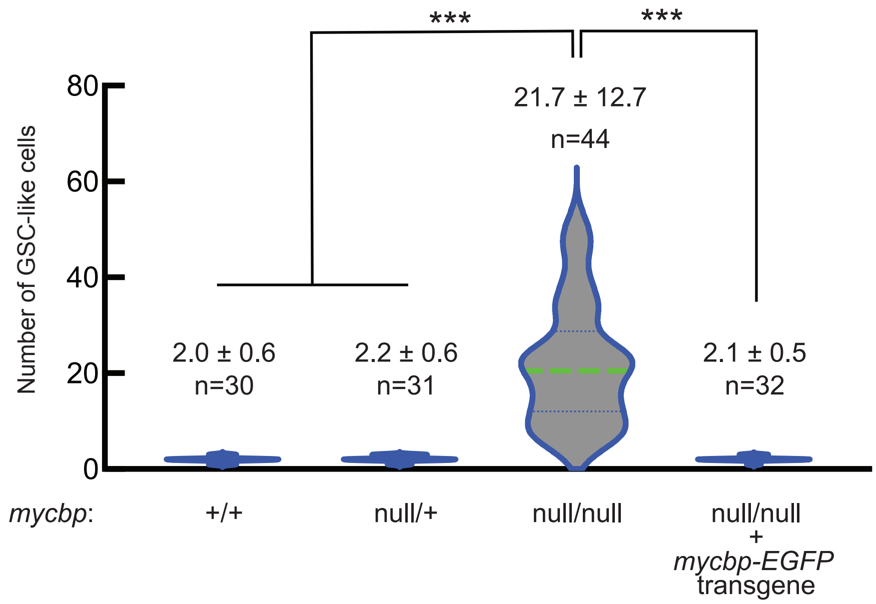

bioRxiv preprint doi: https://doi.org/10.1101/2025.07.01.662550; this version posted July 2, 2025. The copyright holder for this preprint (which was not certified by peer review) is the author/funder, who has granted bioRxiv a license to display the preprint in perpetuity. It is made available under aCC-BY 4.0 International license.

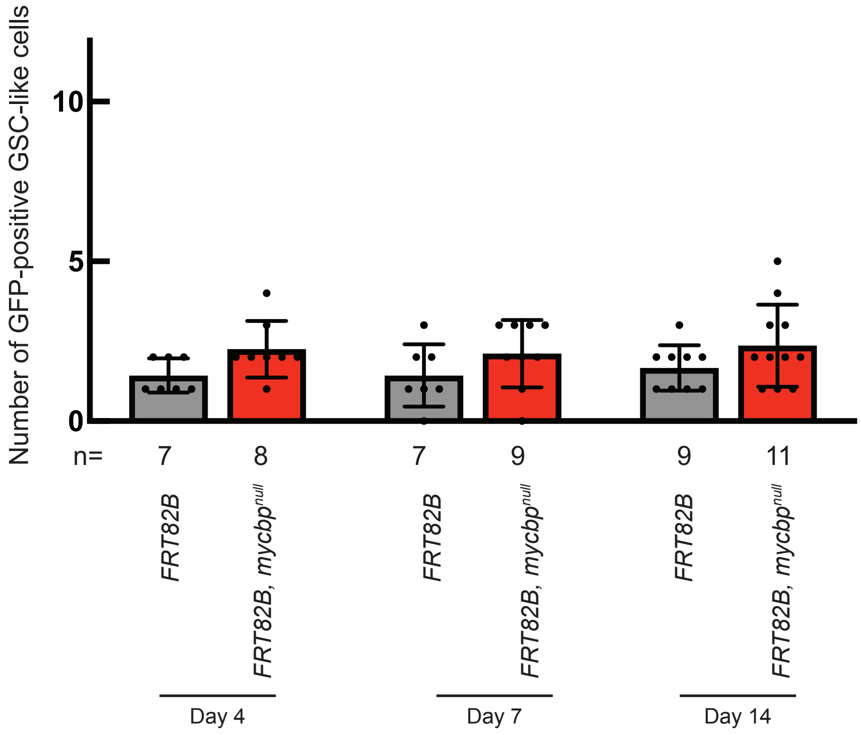

Supplement: Supplement 1 [file NIHPP2025.07.01.662550v1-supplement-1.pdf]
